# Cytoreductive radiotherapy combined with abiraterone in metastatic castration-resistance prostate cancer: a single center experience

**DOI:** 10.1186/s13014-020-01732-y

**Published:** 2021-01-06

**Authors:** Yang Liu, Wen Long, Zitong Zhang, Lixin Mai, Sijuan Huang, Boji Liu, Wufei Cao, Jianhua Wu, Fangjian Zhou, Yonghong Li, Liru He

**Affiliations:** 1 Department of Radiation Oncology, Sun Yat-Sen University Cancer Center, State Key Laboratory of Oncology in South China, Collaborative Innovation Center for Cancer Medicine, 651 Dongfeng Road East, Guangzhou, 510060 People’s Republic of China; 2 Department of Nuclear Medicine, Sun Yat-Sen University Cancer Center, State Key Laboratory of Oncology in South China, Collaborative Innovation Center for Cancer Medicine, Guanghzou, People’s Republic of China; 3 Department of Urology, Sun Yat-Sen University Cancer Center, State Key Laboratory of Oncology in South China, Collaborative Innovation Center for Cancer Medicine, 651 Dongfeng Road East, Guangzhou, 510060 People’s Republic of China

**Keywords:** Metastatic castration-resistant prostate cancer, Cytoreduction, Radiotherapy, Abiraterone

## Abstract

**Background:**

To investigate the potential benefit of cytoreductive radiotherapy (cRT) in metastatic castration-resistant prostate cancer (mCRPC) patients receiving abiraterone.

**Methods:**

From February 2014 to February 2019, 149 mCRPC patients treated with abiraterone were identified. Patients receiving cRT before abiraterone failure (AbiRT group) were matched by one-to-two propensity score to patients without cRT before abiraterone failure (non-AbiRT group).

**Results:**

The median follow-up was 23.5 months. Thirty patients (20.1%) were in the AbiRT group, whereas 119 patients (79.9%) were in the non-AbiRT group. The 2-year OS of patients managed by AbiRT and non-AbiRT were 89.5% and 73.5%, respectively (*P* = 0.0003). On multivariate analysis, only AbiRT (HR 0.17; 95% CI 0.05–0.58; *P* = 0.004) and prognostic index (HR 2.71; 95% CI 1.37–5.35; *P* = 0.004) were significant factors. After matching, AbiRT continued to be associated with improved OS (median OS not reached vs. 44.0 months, *P* = 0.009). Subgroup analysis revealed that patients aged ≤ 65 years (HR 0.09; 95% CI 0.01–0.65; *P* = 0.018), PSA ≤ 20 ng/mL (HR 0.29; 95% CI 0.09–0.99; *P* = 0.048), chemotherapy-naïve upon abiraterone treatment (HR 0.20; 95% CI 0.06–0.66; *P* = 0.008) and in intermediate prognosis groups by COU-AA-301 prognostic index (HR 0.13; 95% CI 0.03–0.57; *P* = 0.007) had improved OS with AbiRT.

**Conclusions:**

cRT before resistance to abiraterone may improve survival in selected mCRPC patients: age ≤ 65 years old, chemotherapy-naïve, with a relatively low PSA level at the diagnosis of mCRPC and intermediate prognosis.

## Background

Prostate cancer is the second most commonly diagnosed cancer in men worldwide [1]. The incidence of metastatic prostate cancer has been increasing, and the number of new cases of metastatic prostate cancer is estimated to increase by 42% over the next decade [2]. Nearly all metastatic prostate cancer will progress into an aggressive state known as metastatic castration-resistant prostate cancer (mCRPC). Without effective treatment, the median OS of these patients is only 9–30 months [3]. In recent years, major advances in therapeutic agents has significantly improved the survival of mCRPC patients. Abiraterone is one of the standard of care for mCRPC. Abiraterone plus prednisone has shown remarkable efficacy and safety in chemotherapy-naïve and chemotherapy-treated mCRPC patients [4, 5]. However, heterogenous responses to abiraterone exist due to the polyclonal nature of metastatic sites, and acquired resistance to abiraterone eventually develop after 6–14 months [4, 5]. Thus, increasing studies are investigating the potential benefit of adding local therapy to systemic therapy in metastatic prostate cancer.

Current cytoreductive treatment strategies for metastatic prostate cancer includes prostate-directed therapy and metastasis-directed therapy. The STAMPEDE trial demonstrates that prostate-directed radiotherapy could prolong survival in metastatic hormone-sensitive prostate cancer (mHSPC) with low metastatic burden [6], and the HORRAD trial also suggests prolonged PSA progression in mHSPC with less than 5 metastases [7]. Beyond local cytoreduction of primary sites, reducing the metastatic burden through local therapy may also bring survival benefit, as observed in ovarian, kidney and some gastrointestinal cancers [8–10]. In prostate cancer, metastasis-directed radiotehrapy is increasingly endorsed in patients with limited metastatic lesions early in their chain of progression. Stereotactic body radiotherapy (SBRT) to all metastatic sites is likely to delay disease progression and prolong systemic in oligometastatic mHSPC, as supported by the results of some phase II trials such as STOMP, ORIOLE and POPSTAR [11–13].

Given the promising results of cytoreductive radiotherapy in mHSPC, we hypothesized that cytoreductive radiotherapy might as well have a role in the event of mCRPC, a more terminal state. In this retrospective study, we sought to investigate the potential benefit of cytoreductive radiotherapy (cRT) in mCRPC patients treated with abiraterone.

## Methods

### Patient selection and baseline evaluation

**Fig. 1:**
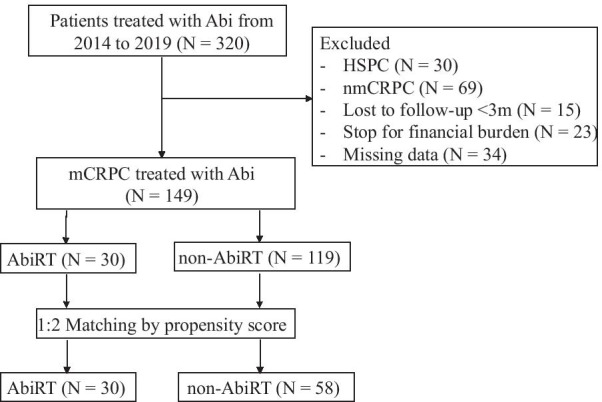
Study flow diagram

This study was approved by the institutional review board. We retrospectively reviewed 320 prostate cancer patients treated with abiraterone plus prednisone between February 2014 and February 2019 in our institution. Inclusion criteria were mCRPC patients receiving abiraterone with or without radiotherapy. Patients were excluded if they were HSPC or nonmetastatic at the time of abiraterone treatment. Patients lost to follow-up less than 3 months after abiraterone treatment, who discontinued abiraterone treatment for financial burden, or who lacked data for risk stratification were also excluded. Finally, a total of 149 patients were included in the analyses (Fig. 1).

Oligometastasis was defined as ≤ 5 metastatic lesions to lymph nodes and/or bones without visceral metastasis [14]. Risk stratification was determined by a prognostic index developed from the COU-AA-301 study [15], which has been validated in both chemotherapy-naive and chemotherapy-treated mCRPC patients [16, 17]. This model comprises six risk factors: lactate dehydrogenase > upper limit of normal (ULN); Eastern Cooperative Oncology Group performance status of 2; liver metastases; albumin ≤ 4 g/dL; alkaline phosphatase > ULN; time from start of initial androgen-deprivation therapy (ADT) to treatment initiation is ≤ 36 months. Patients were categorized into good (0–1 risk factor), intermediate (2–3 risk factors) and poor (4–6 risk factors) prognostic groups.

### Treatment

cRT was prescribed for patients in whom radiotherapy covered the lesions that accounted for > 50% of the total tumor burden [18]. Patients who received cRT could be oligometastatic, or polymetastatic with most of the lesions locating in one region (e.g. pelvis). Radiotherapy to the prostate could be for symptom relief or tumor cytoreduction. Patients treated with cRT before abiraterone failure were placed in the AbiRT group. The comparison group was patients who did not receive cRT before abiraterone failure (non-AbiRT group), including patients receiving cRT after abiraterone failure (cRT + non-Abi) and no cytoreductive RT (non-cRT). All patients had ADT together with 1000 mg of abiraterone once-daily and 5 mg of prednisone twice-daily. Abiraterone was not withheld or its dose was not reduced during radiotherapy.

Primary sites were treated with intensity-modulated radiotherapy, and all metastatic sites were treated with SBRT. Patients underwent simulation with contrast-enhanced CT (slice thickness = 3 mm) with site-specific immobilization. Planning CT images were fused with magnetic resonance images of the skeletal segment interested. Contouring was in accordance with the corresponding recommendations set by the Radiation Therapy Oncology Group (RTOG). For the prostate, the clinical target volume (CTV) included the prostate gland with or without seminal vesicles, and the planning target volume (PTV) was yielded by expansion by 5 mm (3 mm posteriorly). Regional lymph nodes were not contoured unless radiographically positive. Distant metastases were treated with SBRT. The CTV equals the gross tumor volume. The PTV was defined as the CTV plus a margin of ≤ 5 mm.

The prescription dose for the prostate gland and pelvic lymph nodes was 60–67.5 Gy and 45–60 Gy in 25 fractions, respectively. The dosage regimen for metastatic lesions was 18–35 Gy in 1–5 fractions. Dose constraints for normal tissue were in accordance with RTOG guidelines [19]. Treatment was delivered by a linear accelerator using 6–8 MV photons. Image guided radiation therapy using cone-beam CT was performed before each treatment fraction.

### Outcomes

For patients treated with radiotherapy, the PSA tests were carried out 1 month after radiotherapy, and every 3 months thereafter. For patients treated with abiraterone alone, PSA was generally tested every 3 months. Radiological evaluation was ordered at the discretion of physicians. OS was defined as the time from the diagnosis of mCRPC until last follow-up or death from any cause. Progression-free survival (PFS) was measured from the start of radiotherapy until PSA or radiographic progression or death. Biochemical and radiographic progression was assessed according to the Prostate Cancer Clinical Trials Working Group 2 (PCWG2). PSA response was defined as a decline in PSA levels of > 50% from baseline, measured twice 4 weeks apart.

### Statistical analysis

To address the imbalance of potential confounders, we used propensity score matching to compare the OS between the AbiRT and non-AbiRT group. The propensity score was estimated as the probability of receiving AbiRT from a logistic regression model. Variables that were prognostic for OS were explored. The propensity-score model included age, Gleason score, PSA level at mCRPC, prognostic group, synchronous metastasis, oligometastasis, chemotherapy-naïve upon abiraterone treatment. One-to-two matching without replacement was implemented using nearest-neighbor matching. The caliper width was 0.2 times the standard deviation of the logit of the propensity score.

Categorical data were compared using the chi-squared test. OS and PFS were estimated using the Kaplan–Meier method and compared using the log-rank test. The Cox proportional hazard model was used for univariate and multivariate analyses. Variables that were significant in the univariate analysis were included in the multivariate analysis. A *P* value of less than 0.05 was considered significant. All tests were two-sided. Descriptive analysis and survival analyses were carried out by SPSS v23 (IBM, Armonk, NY, USA). Matching of propensity scores was done by Python (www.Python.org).

## Results

The baseline characteristics of the entire cohort are summarized in Table 1. The median PSA level at the time of mCRPC diagnosis was 16.8 ng/mL (range 0.2–3411.0 ng/mL). Seventy-nine patients (53.0%) had a Gleason score of 9 or 10 at diagnosis of prostate cancer. Synchronous metastasis was present in 127 patients (85.2%). Fifty-eight patients (38.9%) had oligometastasis at the time of mCRPC diagnosis. The number of patients allocated to good, intermediate and poor prognostic groups was 65 (43.6%), 75 (50.3%) and 9 (6.0%), respectively. Fifty-four patients received docetaxel (65–75 mg/m^2^) every 3 weeks, with 26 cases (17.4%) before abiraterone treatment. Forty patients (26.8%) underwent cRT, with 12 (30.0%), 10 (25.0%) and 18 (45.0%) patients receiving cRT to prostate, metastatic sites, and both prostate and metastatic sites. Thirty (75.0%) patients were irradiated before abiraterone failure, and the remaining 10 patients (25.0%) received cRT with concurrent secondary hormone therapies after abiraterone failure.

**Table 1:** Baseline characteristics of the entire cohort (N = 149)

Characteristics	No. (%)
Age, median (range), years	68 (45–86)
PSA at mCRPC	
≤ 20 ng/mL	80 (53.7)
> 20 ng/mL	69 (46.3)
Gleason score^a^	
≤ 8	70 (47.0)
9–10	79 (53.0)
ECOG	
0–1	105 (70.5)
> 1	44 (29.5)
Prognostic index^b^	
Good	65 (43.6)
Intermediate	75 (50.3)
Poor	9 (6.0)
Synchronous metastasis	127 (85.2)
Oligometastasis	58 (38.9)
Chemo-naïve	123 (82.6)

*PSA* prostate-specific antigen, *mCRPC* metastatic castration-resistant prostate cancer, *Chemo-naïve* chemotherapy-naïve upon abiraterone treatment

^a^Gleason score at diagnosis of prostate cancer

^b^The COU-AA-301 prognostic index

Thirty patients (20.1%) were in the AbiRT group, whereas 119 patients (79.9%) were in the non-AbiRT group. Compared with the non-AbiRT group, patients in the AbiRT group were more likely to have oligometastasis (*P* = 0.002), with a lower PSA level at mCRPC diagnosis (*P* = 0.005). Other baseline characteristics including age, Gleason score, synchronous metastasis, chemotherapy-naïve upon abiraterone treatment and prognostic index were similar (Table 2).

**Table 2:** Comparison of baseline characteristics in the unmatched and the matched data

Characteristics	Unmatched data			Matched data		
	No. (%)			No. (%)		
	AbiRT (N = 30)	Non-AbiRT (N = 119)	*P*	AbiRT (N = 30)	Non-AbiRT (N = 58)	*P*
Age, years			0.224			0.440
≤ 65	15 (50.0)	45 (37.8)		15 (50.0)	24 (41.3)	
> 65	15 (50.0)	74 (62.2)		15 (50.0)	34 (58.6)	
PSA at mCRPC			0.005			0.667
≤ 20 ng/mL	23 (76.6)	57 (47.9)		23 (76.6)	42 (72.4)	
> 20 ng/mL	7 (23.3)	62 (52.1)		7 (23.3)	16 (27.6)	
Gleason score			0.435			0.886
≤ 8	16 (53.3)	54 (45.4)		16 (53.3)	30 (51.7)	
9–10	14 (46.6)	65 (54.6)		14 (46.6)	28 (48.3)	
Prognostic index			0.107			0.227
Good	17 (56.6)	48 (40.3)		17 (56.6)	25 (43.1)	
Intermediate/poor	13 (43.3)	71 (59.7)		13 (43.3)	33 (56.9)	
Oligometastasis	19 (63.3)	39 (32.8)	0.002	19 (63.3)	36 (62.1)	0.908
Synchronous metastasis	24 (80.0)	103 (86.6)	0.538	24 (80.0)	48 (82.8)	0.750
Chemo-naïve	28 (93.3)	95 (79.8)	0.082	28 (93.3)	53 (91.4)	1.000

*PSA* prostate-specific antigen, *mCRPC* metastatic castration-resistant prostate cancer, *Chemo-naïve* chemotherapy-naïve upon abiraterone treatment

**Fig. 2:**
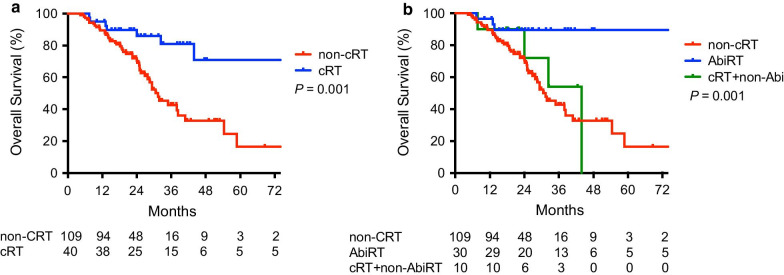
**a** Overall survival for patients with mCRPC treated with (N = 40) and without (N = 109) cytoreductive radiotherapy. **b** Overall survival of patients treated with cytoreductive RT before abiraterone failure (AbiRT, N = 30), after abiraterone failure together with other secondary hormone therapies (cRT + non-Abi, N = 10) and no cytoreductive RT (non-cRT, N = 109)

**Fig. 3:**
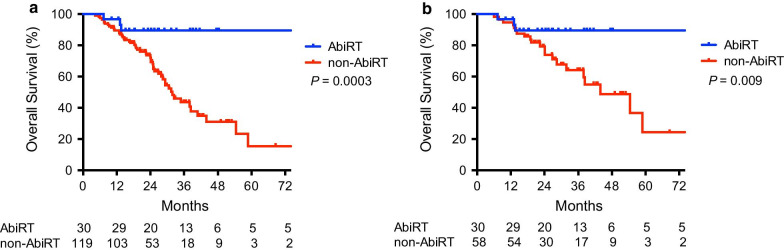
Overall survival for mCRPC patients treated with (AbiRT) and without (non-AbiRT) cytoreductive RT before abiraterone failure before (**a**) and after (**b**) propensity score matching

At a median follow-up of 23.5 months, 54 patients (36.2%) died. Seven patients (4.7%) were lost to follow-up, 3 in the AbiRT group and 4 in the non-AbiRT group. The local control rate following AbiRT was 96.7%. The median OS of the entire cohort was 38.4 months. The median OS of patients undergoing cRT was not reached, compared with 31.4 months in patients who did not have cRT (*P* = 0.001) (Fig. 2). The 2-year OS rates of patients managed by AbiRT, cRT after abiraterone failure, and no cRT was 89.5%, 72.0% and 72.0%, respectively (*P* = 0.001). The median PFS following radiotherapy in the AbiRT group was 12.2 months, and 23 (76.6%) patients had a PSA response after radiotherapy. Chemotherapy was the most frequently chosen treatment (40.0%) after progression, followed by estramustine (15.0%). The median OS of the AbiRT group was not reached whereas, in the non-AbiRT group, the median OS was 31.8 months (*P* = 0.0003). The 2-year OS rates of the AbiRT group and non-AbiRT group were 89.5% and 73.5%, respectively (*P* = 0.0003) (Fig. 3). Upon univariate analysis, AbiRT, oligometastasis, intermediate/poor group according to the prognostic index, PSA > 20 ng/mL and chemotherapy-naïve upon abiraterone treatment were significant prognostic factors for OS (Table 3). Upon multivariate analysis, the AbiRT group [hazard ratio (HR), 0.17; 95% confidence interval (CI), 0.05–0.58; *P* = 0.004] and intermediate/poor grouping for the prognostic index (HR 2.71; 95% CI 1.37–5.35; *P* = 0.004) were significant prognostic factors (Table 3).

**Table 3:** Univariate and multivariable analyses of factors predictive of overall survival

Variables	Univariate analysis		Multivariate analysis	
	HR (95% CI)	*P*	HR (95% CI)	*P*
AbiRT				
Yes versus no	0.15 (0.05, 0.48)	0.001	0.17 (0.05, 0.58)	0.004
Oligometastasis				
Yes versus no	0.52 (0.30, 0.93)	0.028	0.75 (0.39, 1.44)	0.387
PSA > 20 ng/mL				
Yes versus no	2.16 (1.23, 3.79)	0.007	1.66 (0.93, 2.96)	0.089
Prognostic index				
Interm/poor versus favorable	3.11 (1.64, 5.92)	0.001	2.71 (1.37, 5.35)	0.004
Chemo-naïve at Abi				
Yes versus no	0.45 (0.25, 0.81)	0.008	0.81 (0.41, 1.61)	0.548

*PSA* prostate-specific antigen, *Interm* intermediate, *Chemo-naïve* chemotherapy-naïve upon abiraterone treatment, *HR* hazard ratio, *CI* confidential interval

After propensity score matching with a caliper of 0.21, 30 patients in the AbiRT group were matched to 58 patients in the non-AbiRT group. The difference between the baseline characteristics was not significant after matching (Table 2). The survival advantage of AbiRT remained significant. The median OS was not reached in the AbiRT group, compared with 44.0 months in the non-AbiRT group (*P* = 0.009). The 2-year OS of the AbiRT group and non-AbiRT group was 89.5% and 79.3%, respectively (Fig. 3).

**Fig. 4:**
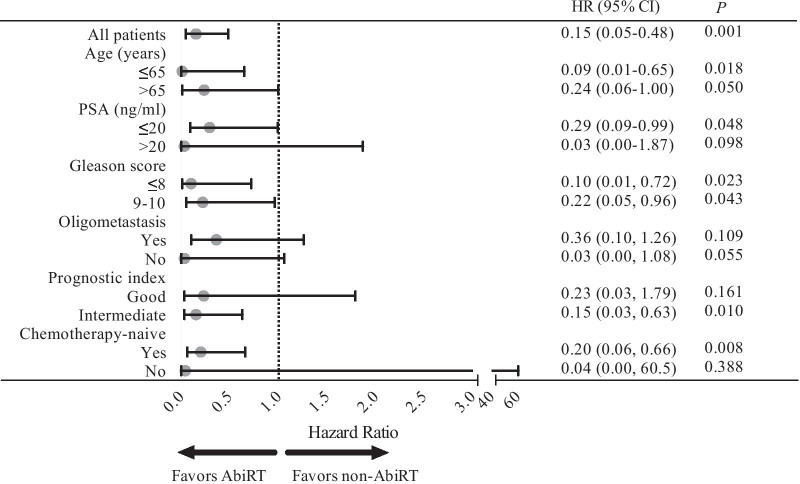
Forest plot of the association between cytoreductive radiotherapy before abiraterone failure and overall survival by subgroup. *HR* hazard ratio, *95% CI* 95% confidence interval

The AbiRT group was associated with improved OS in the subgroups of age ≤ 65 years old, PSA ≤ 20 ng/mL and chemotherapy-naïve upon abiraterone treatment (Fig. 4). The benefit of AbiRT was not evaluated in patients with poor prognosis according to the prognostic index given that there were only nine patients in this subgroup. In the subgroup with an intermediate prognosis according to the prognostic index, application of AbiRT was associated with a reduction of death of 85% (HR 0.15; 95% CI 0.03–0.63; *P* = 0.010); this advantage was not observed in patients with a good prognosis (HR 0.23; 95% CI 0.03–1.79; *P* = 0.161).

Radiotherapy was well tolerated, with no grade 3 or higher toxicities reported. Acute side effects included gastrointestinal (GI) toxicity (9 patients), genitourinary (GU) toxicity (11 patients), thrombocytopenia (1 patient), neutropenia (1 patient) and hypokalemia (1 patient). Late GI and GU toxicity were observed in 7 and 6 patients, respectively. Two patients developed vertebral compression fractures, and neither of them were symptomatic.

## Discussion

Evolving novel hormonal agents have improved the systemic control and prolonged survival of mCRPC patients, and increasing focus is being placed on the potential benefits of cytoreductive local therapy [20]. This study provides valuable insights regarding the value of cytoreductive radiotherapy in mCRPC. In this comparison of survival outcomes between the two groups (AbiRT vs. non-AbiRT), cRT before abiraterone failure was associated with a remarkable improvement in OS, and adoption of cRT required careful consideration when systemic control could no longer be maintained by abiraterone therapy. Patients aged ≤ 65 years with PSA ≤ 20 ng/mL, who were chemotherapy-naïve and belonged to the intermediate prognostic group may be potential candidates for cytoreductive therapies.

Addition of radiotherapy to androgen receptor axis-targeted therapy (ARAT) may provide additional advantages [21]. Androgen-receptor signaling can promote radioresistance by accelerating repair of the DNA damage induced by ionizing radiation [22], and new-generation ARAT can result in downregulation of expression of the DNA repair genes, thereby potentiating the effect of ionizing radiation [23]. In clinical studies, a delay of disease progression has been observed after delivering radiotherapy to mCRPC patients treated with abiraterone [24–26]. Similarly, our study showed that the survival benefit was most pronounced if cRT was combined with abiraterone. Ongoing clinical trials (NCT03449719 and NCT03556904) will help to decide whether combining radiotherapy and abiraterone could provide additional advantage in mCRPC.

cRT can discontinue direct seeding of new metastases as well as stopping supportive interactions between primary and metastatic sites [27, 28]. cRT can also eliminate resistant clones and release a wider range of tumor antigens [29]. Emerging clinical evidence suggests that cRT can significantly prolong PFS and even OS in mHSPC [6, 11–13]. In contrast, cRT for mCRPC seems less attractive because the value of local therapy at such a late stage is questionable. Several retrospective studies show that radiotherapy to oligoprogressive sites could delay disease progression in mCRPC [24, 26, 30]. Yildirim et al. observed no significant improvement of OS was found following prostate-directed radiotherapy in mCRPC (24.1 vs. 21.4 months; *P* = 0.08), while Fujita et al. reported significant improvement of OS (66 vs. 22 months; *P* = 0.001), with 2-year OS around 85% in the prostate-directed radiotherapy group [31]. Our study echoed the finding of Fujita et al., with 2-year OS of 89.5% in the AbiRT group. As half of the patients in the study by Yildirim et al. were post-docetaxel, and the patients in our study and the study of Fujita et al. were predominantly chemotherapy-naïve, it could be speculated that patients are more likely to have survival benefit when cytoreductive local therapy is adopted at an early stage of mCRPC [14]. These results imply that the power of cytoreductive local therapy is restricted at a terminal stage of disease, especially if multiple lines of treatment have failed. Another point to consider is the extent of radiotherapy. The extent of local therapy could affect survival in solid tumors [32, 33]. Previous studies in mCRPC focused exclusively on prostate-directed therapy or oligoprogressive metastases-directed therapy alone. Our patients received greater extent of cytoreduction by radiotherapy when abiraterone was effective, which might account for the encouraging result.

In subgroup analyses, the survival benefit from AbiRT was observed in patients in the intermediate prognostic group instead of in those in the good prognostic group. These data suggested that abiraterone treatment alone could elicit satisfactory control for some low-risk patients, whereas intensive therapy (e.g., AbiRT) was worth trying for intermediate-risk patients because abiraterone treatment alone might be not sufficient for these patients. Interestingly, oligometastasis could not be used to identify potential candidates for local radiotherapy in our study. Current definition of oligometastasis only offers an assessment of tumor burden in a snapshot, but the underlying clinical pathways that lead to an oligomeatstatic state is undefined. The European Society for Radiotherapy and Oncology and European Organisation for Research and Treatment of Cancer has classified the oligometastates into 9 distinct situations [34]. Oligometastasis can be induced by multiple lines of systemic therapies in mCRPC, and patients who are heavily pretreated are not in their early chain of progression despite having limited metastases. These data suggest that oligometastasis, a generally accepted indication for local cytoreductive therapy in newly diagnosed mHSPC, may not be applicable in mCRPC. Thus, the decision regarding local intervention requires combined interpretation of the patient’s general condition, disease state and the profile of systemic treatment [14]. Patients who are not heavily-pretreated, and those who are relatively young with good treatment tolerance as well as a low level of PSA, may benefit from aggressive local therapy in mCRPC.

Our study has several limitations. First, it is a retrospective study. However, given the lack of evidence on the role of cytoreductive radiotherapy in the general situation other than oligometastasis and oligoprogression, our study provides valuable information for current practice. Second, excluding patients with missing data might have led to selection bias (though there is no evidence that these patients were more or less prone to be omitted based on the scarce information in the medical records). Third, our patients represent a heterogenous cohort of mCRPC patients who received different treatment at different time points.

## Conclusions

The present study evaluated the survival outcomes of cytoreductive radiotherapy in mCRPC patients treated with abiraterone. The findings from our study support the use of cytoreductive radiotherapy before the resistance to abiraterone in selected mCRPC patients, preferably in age ≤ 65 years old, chemotherapy-naïve, with PSA ≤ 20 ng/mL at the time of mCRPC and intermediate prognosis.

## Data Availability

The datasets used and/or analyzed during the current study are available from the corresponding author on reasonable request.

## Abbreviations

OS: Overall survival
mCRPC: Metastatic castration-resistant prostate cancer
mHSPC: Metastatic hormone-sensitive prostate cancer
ADT: Androgen-deprivation therapy
SBRT: Stereotactic body radiotherapy
cRT: Cytoreductive radiotherapy
ULN: Upper limit of normal
AbiRT: Cytoreductive radiotherapy before abiraterone failure
non-AbiRT: No cytoreductive radiotherapy before abiraterone failure
RTOG: Radiation Therapy Oncology Group
CTV: Clinical target volume
PTV: Planning target volume
PFS: Progression-free survival
PCWG2: Prostate Cancer Clinical Trials Working Group 2
GI: Gastrointestinal
GU: Genitourinary
